# Effect of an opt‐out point‐of‐care HIV‐1 nucleic acid testing intervention to detect acute and prevalent HIV infection in symptomatic adult outpatients and reduce HIV transmission in Kenya: a randomized controlled trial

**DOI:** 10.1111/hiv.13157

**Published:** 2021-08-25

**Authors:** Eduard J. Sanders, Clara Agutu, Elise van der Elst, Amin Hassan, Evanson Gichuru, Peter Mugo, Carey Farquhar, Joseph B. Babigumira, Steven M. Goodreau, Deven T. Hamilton, Thumbi Ndung'u, Martin Sirengo, Wairimu Chege, Susan M. Graham

**Affiliations:** ^1^ KEMRI ‐ Wellcome Trust Research Programme Kilifi Kenya; ^2^ University of Oxford Headington UK; ^3^ Department of Medicine, Global Health, and Epidemiology University of Washington Seattle WA USA; ^4^ Department of Global Health and Pharmacy University of Washington Seattle WA USA; ^5^ Department of Anthropology and Epidemiology University of Washington Seattle WA USA; ^6^ Center for Studies in Demography & Ecology University of Washington Seattle WA USA; ^7^ Africa Health Research Institute Durban South Africa; ^8^ National AIDS & STI Control Programme Nairobi Kenya; ^9^ National Institutes of Allergy & Infectious Diseases National Institutes of Health Rockville MD USA

**Keywords:** acute HIV infection, diagnostic tests, HIV infection, partner notification, point of care, serology, viral load

## Abstract

**Background:**

In sub‐Saharan Africa, adult outpatients with symptoms of acute infectious illness are not routinely tested for prevalent or acute HIV infection (AHI) when seeking healthcare.

**Methods:**

Adult symptomatic outpatients aged 18–39 years were evaluated by a consensus AHI risk score. Patients with a risk score ≥ 2 and no previous HIV diagnosis were enrolled in a stepped‐wedge trial of opt‐out delivery of point‐of‐care (POC) HIV‐1 nucleic acid testing (NAAT), compared with standard provider‐initiated HIV testing using rapid tests in the observation period. The primary outcome was the number of new diagnoses in each study period. Generalized estimating equations with a log‐binomial link and robust variance estimates were used to account for clustering by health facility. The trial is registered with ClinicalTrials.gov NCT03508908.

**Results:**

Between 2017 and 2020, 13 (0.9%) out of 1374 participants in the observation period and 37 (2.5%) out of 1500 participants in the intervention period were diagnosed with HIV infection. Of the 37 newly diagnosed cases in the intervention period, two (5.4%) had AHI. Participants in the opt‐out intervention had a two‐fold greater odds of being diagnosed with HIV (odds ratio = 2.2, 95% confidence interval: 1.39–3.51) after adjustment for factors imbalanced across study periods.

**Conclusions:**

Among symptomatic adults aged 18–39 years targeted by our POC NAAT intervention, we identified one chronic HIV infection for every 40 patients and one AHI patient for every 750 patients tested. Although AHI yield was low in this population, routinely offered opt‐out testing could diagnose twice as many patients as an approach relying on provider discretion.

## INTRODUCTION

Kenya has the fifth largest HIV epidemic in the world, with an estimated adult population prevalence of 4.9% and 1.3 million adults living with HIV in 2018 [1]. Although 80% of people living with HIV (PLWH) know their status, one in five PLWH remain undiagnosed [1]. HIV testing is the essential first step for the UNAIDS 95‐95‐95 goals to be reached, and strategies to improve HIV case finding should be optimized [2].

Finding undiagnosed PLWH in a declining HIV epidemic is challenging [1, 3]. While conventional facility‐based HIV testing and counselling (HTC) has not achieved high testing coverage in sub‐Saharan Africa [4], facility‐based HIV testing presents several unique opportunities to identify PLWH, offer HIV care and treatment to people newly diagnosed, and ensure that prevention options are extended to partners of PLWH. First, symptomatic people seeking healthcare are concerned about their health and entitled to a diagnosis or the exclusion thereof. Second, providers need to know the HIV status of their patients in order to make appropriate diagnosis, treatment, and management plans. Third, communities in the catchment areas of health facilities are entitled that all is done to mitigate ongoing transmission of a preventable illness, and lastly, healthcare policy‐makers should expect returns on HIV testing policies targeting the population at large.

In sub‐Saharan Africa, provider‐initiated testing and counselling (PITC) is offered to only approximately one in five adult patients seeking healthcare [4]. Challenges impacting uptake of PITC include logistics, data systems and human resources and management – reflecting the weaknesses of health systems in the region [5]. Most patients are willing to be tested [6, 7], but the yield of PITC even in highly endemic areas has recently been estimated at around 1% [3]. Therefore, HIV testing of patients seeking healthcare should be strategic and targeted to increase both the yield and number of newly diagnosed patients.

One weakness, however, of routine rapid tests for HIV diagnosis is that they do not identify patients with acute HIV infection (AHI) [8, 9], which is typically defined as the first weeks after HIV acquisition, during which HIV antibodies are undetectable [10]. Although some AHI patients remain asymptomatic, most experience an acute illness approximately 2 weeks following infection, and the majority of these patients seek healthcare [11, 12, 13, 14, 15]. Common symptoms of AHI include fever, joint and muscle pains, headache and fatigue [16]. Diagnosing AHI is important, as patients with AHI have very high viral loads in the few weeks following acquisition, and their sexual behaviour is unlikely to change until they are diagnosed, linked to HIV care and initiated on antiretroviral therapy (ART) [17, 18]. Acute HIV infection is not easily diagnosed using point‐of‐care (POC) antigen‐antibody assays such as the Determine HIV 1/2 Ab/Ag Combo Rapid Test (Alere, Freehold, NJ, USA), which unfortunately has had inconsistent results and low sensitivity in sub‐Saharan Africa [19, 20], but can be diagnosed using nucleic acid amplification testing (NAAT) or p24‐antigen testing [21].

Previously, we showed that among adults at high risk for HIV‐1 acquisition followed in a research cohort, 69% of those who seroconverted sought healthcare at the research clinic or elsewhere due to AHI symptoms [15]. Although not all patients with AHI develop symptoms compatible with an acute retroviral syndrome [22], AHI symptoms are more common among patients infected with subtype A than subtype C or D, and most HIV infections in Kenya are subtype A [23]. Undiagnosed AHI is associated with substantial risk of onward HIV transmission and a common occurrence overlooked [24]. In two pilot studies conducted by our group in coastal Kenya in 2013 and 2016, we showed that AHI could be detected in  around 1% of symptomatic adults who had negative or discordant HIV rapid test results [25, 26]. For the present study, we used a consensus AHI risk score derived from pooled data from Kenya, Malawi and South Africa [16]. With the recent availability of POC HIV‐1 NAAT platforms in sub‐Saharan Africa, real‐time AHI testing before the patient leaves the clinic has become feasible [27], and could be used to supplement opt‐out testing approaches in health facilities.

In the present proof‐of‐concept study, we evaluated the yield of a targeted HIV‐1 testing intervention offered to adults aged 18–39 years who were seeking healthcare at four public and two private health facilities in coastal Kenya, before and after intervention delivery, using a modified stepped‐wedge trial design. The HIV‐1 testing intervention consisted of opt‐out POC HIV‐1 NAAT followed by standard rapid antibody tests to distinguish AHI from chronic HIV among all adult outpatients presenting for healthcare who met our AHI risk score and agreed to enrol [16]. HIV‐1 testing in the observation period consisted of PITC using standard rapid tests without additional support to facility staff for implementation. The primary outcome was new HIV diagnoses in each study period. Secondary outcomes included linkage to HIV care and ART initiation among newly diagnosed index participants, and the yield of HIV partner notification (HPN) using the HIV‐1 testing intervention, when offered to their partners [28]. Our objective with the present analysis was to compare primary and secondary outcomes across study periods (pre‐ and post‐intervention).

## METHODS

### Study setting

**FIGURE 1:**
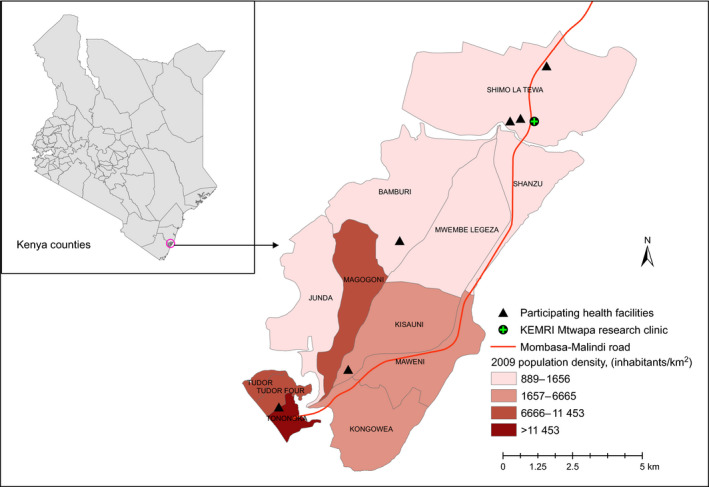
Map of Tambua Mapema Plus study sites [Colour figure can be viewed at wileyonlinelibrary.com]

We conducted our study at four public and two private health facilities in a peri‐urban area (population > 100 000) in Kilifi and Mombasa County, coastal Kenya (Figure 1). HIV prevalence estimates in Kilifi and Mombasa counties in 2018 were 2.3% and 5.6%, respectively [1]. This area has been the site of a KEMRI HIV/Sexually Transmitted Infection (STI) Research Clinic since 2005. We identified the participating health facilities (three in Kilifi county; three in Mombasa county) due to their size, location (within 20 km of our KEMRI Research Clinic), patient volume (> 500 patients aged 18–39 years seen over 3 months) and willingness to collaborate with the research team. All clinics served general population patients; the four public facilities included offered limited programming for key populations.

### Study design

We used a modified stepped‐wedge design to evaluate the yield of the HIV‐1 NAAT intervention, before (1375 patients) and after (1500 patients) intervention delivery [28]. We chose a modified stepped‐wedge trial design as the intervention would do more good than harm and it was not practical to deliver the intervention simultaneously to all participants, due to resource constraints. Each phase lasted 6 months per site, with the exception of the first site having only 3 months of observation [28]. The primary outcome was a new HIV diagnosis, combining chronic HIV and AHI diagnoses.

### Study population

Eligibility criteria for participation included: age 18–39 years; not previously diagnosed with HIV infection; and a score ≥ 2 on our published AHI risk score algorithm [16], with scoring as follows: age 18–29 years (1), fever (1), fatigue (1), body pains (1), diarrhoea (1), sore throat (1) and genital ulcer disease (GUD) (3). Patients who agreed to screening and were eligible were offered enrolment after consenting by the research team.

### Observation period

In the observation period, HIV testing was only done if ordered by the primary care clinician and was carried out using HIV rapid testing per Kenyan Ministry of Health guidelines [29]. Up to 20 participants were targeted for enrolment each week (maximum of four per day). Following the clinical consultation and prior to HIV testing (if ordered), research procedures in the observation period consisted of a computer‐assisted self‐interview (CASI)/computer‐assisted personal interview (CAPI) to assess sociodemographic characteristics, HIV testing history and sexual risk behaviour. Participants found to be HIV‐negative and those not tested ended their participation at the baseline visit. Those newly diagnosed with HIV had a follow‐up home visit at 6 weeks, which included a second CASI/CAPI assessing linkage to HIV care and treatment, as well as partner outcomes. Those who had not yet notified partners at this time point were offered standard HPN services [30].

### Intervention period

Screening and enrolment procedures were similar as in the observation period. However, all enrolled participants were routinely offered the POC HIV‐1 NAAT, followed by two rapid HIV tests when the X‐pert was positive (details below). Test results were provided to participants in real time, with post‐test counselling by research staff. Newly diagnosed intervention period study participants were offered enrolment in an HIV care cohort at the nearby KEMRI Research Clinic and encouraged to start government‐provided ART upon linkage (the same day as diagnosis or as soon as possible). These index patients were asked about sexual partners in the past year (chronic HIV infection) or the past 3 months (AHI) and offered enhanced HPN, in which the HIV‐1 NAAT intervention was used to test all partners successfully reached. Index participants judged to be at moderate or high risk of intimate partner violence were excluded from enhanced HPN. Partners who tested positive were offered enrolment in the KEMRI ART cohort, while those who tested negative and were in an ongoing relationship with the index participant were offered enrolment into a pre‐exposure prophylaxis (PrEP) cohort. Follow‐up in the ART and PrEP cohorts was for 12 months.

### Laboratory testing

A 4 mL blood sample was obtained and tested using the Xpert^®^ HIV Qual assay (GeneXpert^®^ HIV‐1 Qual Cepheid, Sunnyvale, CA, USA) on‐site at the health facility where the participant was recruited and enrolled. This assay has been found to be easy to use and feasible in a community‐based facility with limited or no laboratory infrastructure [24]. For participants in whom HIV‐1 nucleic acid was detected, a laboratory technician conducted rapid antibody testing (currently Determine^®^, Abbott Laboratories; and First Response^®^ (Mumbai, India), Premier Medical Corporation, Princeton, NJ, USA) in accordance with Kenyan HIV testing guidelines [29], to distinguish acute (nucleic acid‐positive, antibody‐negative or indeterminate) from chronic (antibody‐positive) HIV infection. This algorithm obviated the need to conduct both rapid antibody tests and Xpert HIV‐1 Qual test on the vast majority of samples, reducing overall test costs.

### Statistical analysis

We compared the number and reasons of individuals who accepted *vs*. refused screening in the observation *vs*. intervention phase, the number and reasons for screening out participants in each phase, and, among those eligible, the number and reasons for refusing study participation in each phase, using χ^2^ or Fisher’s exact tests for categorical variables as appropriate. We compared the proportion of patients with the following outcomes in the observation and intervention periods: (1) tested for HIV infection; (2) newly diagnosed with chronic HIV infection (i.e. HIV seropositive); and (3) newly diagnosed with AHI.

We conducted analyses on the individual‐level data, using generalized estimating equation (GEE) models to account for clustering by health facility, with a log link, binomial distribution, and robust variance estimates [29]. Using GEE, we first compared the primary outcome (i.e. new HIV diagnoses) across arms for an unadjusted estimate. We next compared baseline characteristics between individuals in the observation and intervention groups to identify imbalances across study periods. Where imbalances were identified (i.e. differences significant at *P* < 0.20), we controlled for these potential confounders in secondary analyses using GEE models of the primary outcome as described earlier. We compared the age of newly diagnosed cases by Wilcoxson rank‐sum test in the intervention and observation periods.

### Ethical considerations

The study received ethical approval by the KEMRI Scientific and Ethical Review Unit (KEMRI/SERU/CGMRC‐C/051/3280), the Human Subjects Division at the University of Washington (STUDY00001808), and the Oxford Tropical Research Ethics Committee (OxTREC) at the University of Oxford (reference: 46‐16). The protocol was approved by the Division of AIDS (DAIDS), National Institute of Allergy and Infectious Diseases (NIAID), andn the National Institutes of Health (NIH) (DAIDS‐ES 38181).

## RESULTS

**FIGURE 2:**
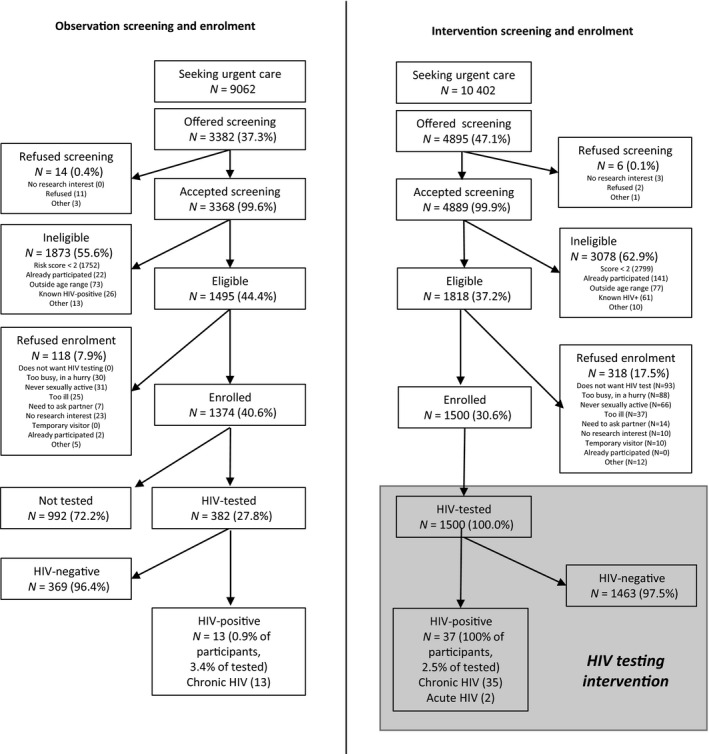
Consort diagram: Tambua Mapema Plus Trial profile. Five (4.2%) and twelve (3.7%) participants in the observation and intervention phases, respectively, provided more than one reason when they refused research participation

A total of 19 464 patients aged 18–39 years sought healthcare at one of the six study health facilities during the period December 2017 to March 2020: 9062 patients in the observation period and 10 402 in the intervention period (Figure 2). Slightly more patients refused study screening in the observation period (0.4%) compared with the intervention period (0.1%, *P* = 0.008). Most patients (59.9%) who were ineligible had a risk score < 2, while being outside the age range, prior HIV diagnosis, and previous participation in the study were less common reasons for ineligibility. More eligible patients refused enrolment in the intervention period (318/1818 or 17.5%), than in the observation period (118/1495 or 7.9%, *P* < 0.001), with notable increases in refusal due to HIV testing (29.3% of eligible intervention period patients *vs*. no eligible observation period patients).

In total, 3374 participants enrolled: 1374 in the observation period and 1500 in the intervention period (Table 1). Overall, 61.7% of participants were female, 40.6% were employed, and the median (interquartile range, IQR) age was 25 (23–30) years. The frequency of reported symptoms was as follows: fever (48.1%), diarrhoea (15.2%), fatigue (72.1%), body pains (66.1%), sore throat (28.7%) and genital ulcers (7.1%). Among those who were sexually active in the last 6 weeks (69.9%), 27.5% of participants reported unprotected sex with a partner of unknown HIV status and 7.8% reported multiple partners. Among men, 0.2% reported same‐sex behaviour.

**TABLE 1:** Characteristics of enrolled participants, Tambua Mapema Plus Trial, coastal Kenya, 2017–2020

	Total (*N* = 2874)	Observation phase (*N* = 1374^a^)	Intervention phase, (*N* = 1500)	*P*‐value
Sex				
Male	1101 (38.3)	488 (35.5)	613 (40.9)	**0.088**
Female	1773 (61.7)	886 (64.5)	887 (59.1)	
Age (median, IQR)	25 (23, 30)	25 (23, 29)	26 (23, 30)	**0.143**
Education level^a^				
Primary or below	1102 (38.4)	513 (37.4)	589 (39.3)	0.611
Secondary	1068 (37.2)	542 (39.6)	526 (35.1)	
Tertiary	699 (24.4)	315 (23.0)	384 (25.6)	
Marital status^a^				
Single	1302 (45.4)	649 (47.4)	653 (43.6)	0.402
Married	1394 (48.6)	635 (46.3)	759 (50.6)	
Separated/divorced/widowed	173 (6.0)	86 (6.3)	87 (5.8)	
Religion^a^				
Christian	2216 (77.6)	1053 (76.9)	1163 (77.6)	0.484
Muslim	608 (21.2)	295 (21.5)	313 (20.9)	
None	45 (1.6)	22 (1.6)	23 (1.5)	
Source of income^a^				
Employed	1164 (40.6)	529 (38.6)	635 (42.4)	**0.046**
Self‐employed (casual)	768 (26.8)	364 (26.6)	404 (26.9)	
Unemployed	937 (32.7)	477 (34.8)	460 (30.7)	
Symptoms reported				
Fever	1383 (48.1)	663 (48.3)	720 (48.0)	0.955
Diarrhoea	437 (15.2)	195 (14.2)	242 (16.1)	0.261
Fatigue	2072 (72.1)	904 (65.8)	1168 (77.9)	**< 0.001**
Body aches	1899 (66.1)	886 (64.5)	1013 (67.5)	**0.185**
Sore throat	824 (28.7)	376 (27.4)	448 (29.9)	0.401
Genital ulcer	204 (7.1)	91 (6.6)	113 (7.5)	0.653
Sexual risk behaviour				
Risk group^a^				
Heterosexuals	1948 (67.9)	897 (65.5)	1051 (70.1)	0.842
Key populations	61 (2.1)	19 (1.4)	42 (2.8)	
Populations not sexually active in past 6 weeks	860 (30.0)	454 (33.1)	406 (27.1)	
Unprotected sex with partner of unknown HIV status^b^	788 (27.5)	277 (20.2)	511 (34.1)	**< 0.001**
Unprotected sex with partner of known HIV‐positive status^b^	26 (0.9)	5 (0.4)	21 (1.4)	**0.001**
Multiple sex partners^b^	223 (7.8)	76 (5.5)	147 (9.8)	**< 0.001**
Transactional sex^b^	52 (1.8)	15 (1.1)	37 (2.5)	**< 0.001**
Male same‐sex behaviour^b^	7 (0.2)	3 (0.2)	4 (0.3)	0.833
People who inject drugs^b^	3 (0.1)	1 (0.1)	2 (0.1)	0.543
HIV testing history				
Never tested for HIV	300 (10.4)	147 (10.7)	153 (10.2)	0.820
Tested for HIV in last year	1062 (37.0)	499 (36.3)	563 (37.5)	0.649
Tested for HIV in last 3 months	404 (14.1)	234 (17.0)	170 (11.3)	**0.018**

Abbreviation: IQR, interquartile range.

^a^ One participant who enrolled twice excluded after closure of the observation phase.

^b^ Missing in five participants (four observation, one intervention).

In the observation phase, 352 (25.6%) participants were tested for HIV based on orders from a primary care clinician, of whom 13 (3.7%, or 0.9% of all observation study participants) were newly diagnosed with chronic HIV infection. In the intervention phase, all participants were tested for HIV, of whom 37 (2.5%) participants were newly diagnosed with HIV, including 35 (2.3%) with chronic HIV infection and two (0.1%) with AHI (Figure 2). The yield of observational testing (3.7%) was not statistically significantly different from the yield of intervention testing (2.5%, *P* = 0.2). The unadjusted odds of an HIV diagnosis in the intervention period were 2.71 [95% confidence interval (CI): 1.66–4.33, *P* < 0.001).

Characteristics that differed in the observation and intervention periods at *P* < 0.10 included sex, source of income, reported fatigue, sexual risk behaviour and history of HIV testing. Compared with the observational period, fewer participants in the HIV‐1 testing intervention period were female (59.1% *vs*. 64.5%); more were employed (42.4% *vs*. 38.6%) and more reported fatigue (77.9% *vs*. 65.8%). In addition, more intervention participants reported sex with a partner of unknown status (34.1% *vs*. 20.2%), sex with a known‐positive partner (1.4% *vs*. 0.4%), multiple partners (9.8% *vs*. 5.5%) and transactional sex (2.5% *vs*. 1.1%).

After adjustment for factors that were imbalanced across study periods, the odds of an HIV diagnosis in the intervention period were 2.21 (95% CI: 1.39–3.51, *P* = 0.001). Of note, the female to male ratios of newly diagnosed index participants in the observation and intervention periods were similar (6/7 *vs*. 20/17, *P* = 0.98). The median ages of newly diagnosed women in the observation *vs*. the intervention period was statistically significantly different (33.5 *vs*. 26.2, *P* = 0.03); the median ages for newly diagnosed men across the two periods were not different (32.1 *vs*. 33.6, *P* = 0.39).

At the visit 6 weeks after diagnosis, 33 (89.2%) of 37 intervention index participants *vs*. nine (69.2%, *P* = 0.05, one‐sided) out of 13 observation index participants were successfully linked to HIV care and had initiated ART. Of the 30 intervention index participants who enrolled for HIV care at the KEMRI HIV/STI Research Clinic (81.1% of the total), 16 (53.3%) agreed to enhanced HPN.

Ten partners (27.0%) of 37 index participants were tested in the intervention period, compared with three partners (23.1%, *P* = 0.78) of 13 index participants in the observation period. Of these, two intervention period partners (2/10, or 20.0%) and two observation period partners (2/3, or 66.7%, *P* = 0.12) were newly diagnosed with chronic HIV infection. No intervention period partners were diagnosed with AHI. Details of the newly diagnosed cases in the intervention period, their viral load at diagnosis, and their linkage and partner notification outcomes are presented in Table 2; detailed data were not available for observation period cases, as they were referred to the clinic at which they were diagnosed. Four regular male partners of index participants started PrEP in the intervention period research cohort. No partner started PrEP in the observational period, when clinics delivered standard care.

**TABLE 2:** Characteristics of intervention period index participants and partner testing outcomes, Tambua Mapema Plus Trial, coastal Kenya, 2017–2020

No.	Sex	Age (years)	Chronic or acute HIV infection	Linkage outcome	Log_10_viral load	Regular partners reported	Casual partners reported	Consented to HPN	Positive tests/no. tested	Partner HIV status and linkage outcome
1	Female	19	Acute	Enrolled	ND^a^	1	0	Yes	0/1	Spouse confirmed negative and initiated on PrEP
2	Female	23	Chronic	Enrolled	4.74	2	1	Yes	0/1	Spouse confirmed negative and initiated on PrEP; other regular partner status unknown; casual partner status unknown
3	Female	23	Chronic	Other^b^	N/A	1	0	N/A		Status unknown
4	Female	24	Chronic	Enrolled	2.96	1	0	Yes	0/1	Spouse confirmed negative and initiated on PrEP
5	Female	24	Chronic	Enrolled	ND^c^	1	0	No		Status unknown
6	Female	25	Chronic	Not enrolled	Not done	Not assessed	Not assessed	N/A		N/A
7	Female	26	Chronic	Enrolled	5.13	1	0	Yes		Status unknown
8	Female	26	Chronic	Enrolled	5.01	2	0	Yes	0/1	Regular partner confirmed negative initiated on PrEP; spouse status unknown
9	Female	26	Chronic	Enrolled	6.06	1	0	Yes		Status unknown
10	Female	26	Acute	Enrolled	6.91	1	0	No		Regular partner tested positive first, linked at testing facility. Index case referred to study team with AHI symptoms
11	Female	26	Chronic	Enrolled	6.17	1	5	Yes	0/2	Spouse status unknown; two casual partners confirmed negative (not in an ongoing relationship); three other casual partners status unknown
12	Female	27	Chronic	Enrolled	4.22	1	0	Yes	0/1	Regular partner confirmed negative, not in an ongoing relationship
13	Female	27	Chronic	Enrolled	4.81	1	0	No		Status unknown
14	Female	28	Chronic	Enrolled	6.42	1	0	No		Spouse reported to be known positive and on treatment
15	Female	28	Chronic	Enrolled	4.69	0	0	No		Regular partner deceased
16	Female	31	Chronic	Enrolled	4.73	1	1	Yes		Regular partner and casual partner status unknown
17	Female	31	Chronic	Enrolled	3.60	1	5	Passive referral	0/1	Spouse confirmed negative initiated on PEP^d^; casual partners' status unknown
18	Female	32	Chronic	Enrolled	4.23	1	0	Passive referral	1/1	Regular partner newly diagnosed with chronic HIV
19	Female	34	Chronic	Enrolled	4.71	1	0	No		Status unknown
20	Female	39	Chronic	Enrolled	5.05	1	0	Yes		Spouse reported to have tested elsewhere (self‐reported negative)
21	Male	20	Chronic	Enrolled	4.85	1	0	No		Status unknown
22	Male	24	Chronic	Enrolled	4.76	1	0	Yes		Spouse reported to be known positive and on treatment
23	Male	24	Chronic	Enrolled	4.50	2	2	Yes		Spouse tested elsewhere (self‐reported negative); other regular partner status unknown; casual partners' status unknown
24	Male	25	Chronic	LTFU	6.16	1	0	No		Status unknown
25	Male	29	Chronic	Enrolled	3.72	1	8	Yes		Regular partner status unknown; casual partners' status unknown
26	Male	30	Chronic	Enrolled	4.90	1	0	No		Regular partner reported to be known positive and on treatment
27	Male	31	Chronic	Not enrolled	Not done	2	0	N/A		Spouse reported to be known positive and on treatment; other regular partner status unknown
28	Male	31	Chronic	Enrolled	6.58	1	1	No		Spouse and casual partner status unknown
29	Male	34	Chronic	Not enrolled	Not done	Not assessed	Not assessed	N/A		N/A
30	Male	36	Chronic	Enrolled	3.20	2	2	Yes		Spouse tested elsewhere (self‐reported negative); other regular partners' status unknown; casual partners' status unknown
31	Male	36	Chronic	Enrolled	5.73	0	0	No		N/A
32	Male	36	Chronic	Enrolled	4.21	2	0	Yes		Spouse tested elsewhere (self‐reported negative); other regular partner known positive and on treatment
33	Male	36	Chronic	Enrolled	4.01	1	0	No		N/A
34	Male	38	Chronic	Other^e^	N/A	2	0	Passive referral		One spouse reported to be a known positive. Second spouse newly diagnosed with chronic HIV and linked at facility of index enrolment
35	Male	39	Chronic	Other^e^, ^f^	N/A	Not assessed	Not assessed	N/A		N/A
36	Male	39	Chronic	Enrolled	5.47	0	1	No		Casual partner status unknown
37	Male	39	Chronic	Enrolled	4.14	2	0	Yes	1/1	Regular partner newly diagnosed with chronic HIV and initiated on ART; spouse status unknown

Abbreviations: HPN, HIV partner notification; LTFU, lost to follow‐up; ND, not detected; PrEP, pre‐exposure prophylaxis.

^a^ Elite controller.

^b^ Relocated outside of study area.

^c^ Participant denied prior HIV testing or use of ART (CD4 count at diagnosis was 168 cells/mL).

^d^ Participant initiated on post‐exposure prophylaxis (PEP) with HIV exposure < 72 h; no PrEP continuation because of COVID‐19 lockdown.

^e^ Linked to care at testing facility.

^f^ Participant linked to care at testing facility but died before week 6 visit.

## DISCUSSION

This study evaluated a targeted HIV‐1 NAAT intervention to detect acute and chronic HIV infection that was systematically offered to adult outpatients aged 18–39 years seeking healthcare for symptoms compatible with AHI. Implementation of this intervention resulted in a 2.2‐fold higher odds of new HIV diagnosis among participants in the intervention period compared with the observation period, in which standard PITC was conducted, with testing ordered by the primary care clinician. In addition, intensive efforts to link patients to HIV care and expedite ART initiation, test partners and link partners to treatment or prevention as indicated were successful and may have improved clinical outcomes by decreasing time to viral suppression among participants living with HIV and protecting their HIV‐negative partners in ongoing relationships via PrEP initiation.

Our study aimed to increase the number of new HIV diagnoses by consistently implementing a targeted opt‐out HIV testing approach using the GeneXpert^®^ HIV‐1 Qual assay as a POC test. All patients willing to enrol in the intervention period were tested by a team whose procedures were incorporated into the patient flow. This intervention took the burden off the provider to recommend testing and ensured that all participants who were willing to be tested would leave the facility knowing their status. Provider‐initiated testing and counselling are infrequently offered in African health facilities for various logistical reasons, including high patient load, time constraints, staff shortages and fluctuations in test kit supplies [31]. While our intervention required additional resources, our study provides proof‐of‐concept that most Kenyan patients are willing to be tested and can be linked to HIV care and partner notification within facilities, provided a system is set up to ensure HIV testing. Offering oral HIV self‐testing to patients presenting with symptoms of acute infectious illness would be one approach to increase feasibility; this strategy has proven effective in settings similar to ours [6, 32]. While oral HIV self‐testing would not identify AHI, if applied in our study, it would have identified one chronic HIV infection for every 40 participants tested in the intervention period.

In the present study, we found only two AHI cases, suggesting that AHI is uncommon in the population studied. In an earlier study conducted in a different town in coastal Kenya in 2016, targeted opt‐out HIV‐1 NAAT supported by rapid antibody testing led to detection of 24 (3.4%) chronic HIV‐1 infections and six (0.9%) acute HIV infections among 706 care‐seeking participants aged 18–35 years who were identified using the same AHI risk score algorithm as in the present study, for a 25% increase in new HIV diagnoses [26]. The 0.9% AHI prevalence in our pilot study was close to the 1% AHI prevalence among sexually transmitted diseases patients reported by Rutstein *et al*. [33] in Malawi, using a risk score algorithm that combined sexual risk behaviour (i.e. more than one partner in the previous 2 months), symptoms including GUD, and discordant rapid test results [34]. However, in the present study, AHI prevalence was much lower, at only 0.1%, and HIV‐1 NAAT increased the number of new HIV diagnoses by only 6% (from 35 to 37 cases). It is of interest that our engagement with providers in the community led to referral of a symptomatic woman whose male partner had just been diagnosed with chronic HIV for AHI screening, demonstrating that knowledge of AHI and available HIV‐1 NAAT could be helpful in the right clinical situation. As the availability of Xpert machines increases for other clinical uses (e.g. HIV viral load monitoring, TB diagnosis), use of the Xpert HIV‐1 Qual or Quant assays for the purpose of AHI diagnosis may become more common [35].

Although the yield of PITC among those tested in the observation phase (3.7%) was not significantly different from the yield of HIV testing (2.5%) among all participants in the intervention phase, it suggests that healthcare providers target patients who are more likely to have HIV. In an analysis of who was offered PITC in the observation period, healthcare providers more often offered PITC to older participants, men, those who had tested > 1 year ago or never tested, and participants with certain symptoms (i.e. sore throat or ulcer disease) [7]. Because providers tested only about one in four patients seeking healthcare in the observation period, they probably missed a substantial number of cases, especially among younger women, who made up a greater proportion of the cases diagnosed in the intervention period.

While screening acceptability was similar among both arms, more eligible patients (17.5% vs. 7.9%, *P* < 0.001) refused enrolment in the intervention period, and 29.2% of intervention period refusals were due to the HIV testing offered. Refusals in the observation period more often occurred because patients were not interested in research participation or felt too ill. Of interest, the intervention period population included more participants who reported risky sexual behaviour (e.g. unprotected sex with a partner of unknown status, or multiple sex partners) than participants in the observation period, suggesting that patients with a higher risk perception may have been more likely to enrol when testing was presented as a standard component of the study.

In this trial of an HIV‐1 NAAT intervention, we identified one AHI case for 750 participants meeting our age and symptom criteria algorithm. Planned modelling and cost‐effectiveness work will help to elucidate the potential impact of opt‐out AHI detection as done in this study, as compared with opt‐out HIV testing using standard rapid tests, on the Kenyan HIV epidemic and the situations in which such testing would be cost‐effective. We note that our testing intervention and intensive linkage to HIV care with HPN were conducted by a team of researchers fully committed to this work, representing a substantial investment of personnel and other resources. In reality, HIV is only one of the many problems that patients may present with, patient numbers at many public and private health facilities are large, and healthcare providers may prefer testing options that take less time or are conducted by patients themselves using approaches such as HIV oral self‐testing [32].

Our study had several limitations. First, our offer of HIV testing to all intervention participants as part of study procedures led to differences in the study population enrolled in each period. Second, we did not obtain a blood sample from all observation period participants, and were therefore unable to assess the number of acute and chronic HIV infections missed by standard care. Third, we did not collect data on the reasons providers offered PITC to specific patients. Fourth, our study sample had only a small proportion (< 3%) of individuals from key populations at higher risk for HIV infection, such as men who have sex with men, male and female sex workers, and people who inject drugs. Fifth, our study will have missed patients with no or few symptoms who actually had AHI, as they would not have met our eligibility criteria. Lastly, we did not exclude participants who were not sexually active in the 6 weeks prior to study screening (one‐third of the study population), who would therefore not have been at risk for AHI.

Despite these limitations, our study is the first study in Africa to have tested the effect of an opt‐out HIV‐1 NAAT intervention to diagnose both acute and chronic HIV infections among care‐seeking outpatients. We have shown that while AHI was very uncommon, this intervention was very acceptable to patients and increased the odds of a new HIV diagnosis by over two‐fold. Linkage to immediate treatment and HPN, with PrEP provision to uninfected partners was also acceptable and efficient when done by the same team. Planned modelling and cost‐effectiveness evaluations will help in evaluating the potential impact of such an approach. Regardless, health facilities are a promising and important site for identification patients with undiagnosed HIV infection.

## Data Availability

SMG and EJS designed the trial, wrote the protocol, provided study oversight, conducted data analysis, and prepared the draft manuscript. CA led the trial implementation. All authors reviewed and contributed to the final manuscript.
